# Anthropometry of the proximal femur and femoral head in children/adolescents using three-dimensional computed tomography-based measurements

**DOI:** 10.1007/s00276-021-02841-3

**Published:** 2021-10-01

**Authors:** Ali Darwich, Christiane Geiselhardt, Mohamad Bdeir, Sonja Janssen, Stefan O. Schoenberg, Sascha Gravius, Ahmed Jawhar

**Affiliations:** 1 Department of Orthopaedics and Traumatology Surgery, University Medical Centre Mannheim, Medical Faculty Mannheim, Heidelberg University, Theodor-Kutzer-Ufer 1-3, 68167 Mannheim, Germany; 2 Clinic of Radiology and Nuclear Medicine, University Medical Centre Mannheim, Medical Faculty Mannheim, Heidelberg University, Theodor-Kutzer-Ufer 1-3, 68167 Mannheim, Germany; 3 Department of Trauma, Orthopedics, Hand and Reconstructive Surgery, Klinikum Worms, Academic Teaching Hospital of the University Mainz, Gabriel-von-Seidl-Straße 81, 67550 Worms, Germany

**Keywords:** Anthropometry, 3D computed tomography, Measurements, Proximal femur, Children

## Abstract

**Purpose:**

Defining normal anthropometric ranges of proximal femur and femoral head for each age group in children/adolescents is a necessity when differentiating normal anatomical variants from pathological deformities. Aim of this study is to define a set of normal anthropometric parameters based on 3D-CT measurements in normal asymptomatic children/adolescents and analyse the variations arising depending on age, side, and/or gender.

**Methods:**

Morphology of the proximal femur was retrospectively assessed in 170 hips (85 children, < 15 years). Measurements included covered femoral head volume (CFHV), femoral head diameter (FHD), femoral head extrusion index (FHEI), coronal alpha angle (CAA), lateral centre-edge angle (LCEA), anterior (AOS) and posterior head-neck offset (POS) and femoral neck-shaft angle (FNSA). Correlation analyses as well as inter- and intra-rater reliability were performed.

**Results:**

CFHV, LCEA, FHD and AOS/POS increased with age and FHEI, CAA, and FNSA decreased with age. None of the measurements correlated with the side. AOS showed a poor correlation with gender. Rapid growth phases were observed at the age of 1, 7 and 11. The inter- and intra-rater reliability was high (range ICC 0.8–0.99 Cronbach alpha 0.86–0.99).

**Conclusion:**

This data delivers a description of growth phases as well as gender and age-correlated reference values of the proximal femoral morphology that could be used by paediatricians and orthopaedic/paediatric surgeons to early diagnose proximal femur deformities and provide guidance in the planning of possible operations.

**Supplementary Information:**

The online version contains supplementary material available at 10.1007/s00276-021-02841-3.

## Introduction

The hip joint is considered as a ball-and-socket joint between the acetabulum and the proximal femur and femoral head facilitating load transmission to the lower limbs. It is the second largest load-bearing joint in the body, following the knee joint [1].

The hip may show a large array of morphological variations, especially with increasing age or depending on gender. These variations can sometimes be excessive and pathological leading to a painful unproportionate load distribution in the joint causing eventually pathological wear of the joint cartilage, thus being defined as biomechanical risk factors for osteoarthritis [3]. The most prevalent pre-arthrotic condition is by far hip dysplasia, which represents a typical altered distribution of load causing focal cartilage damage [16]. Children with developmental dysplasia of the hip (DDH) present not only with acetabular malformation and hypoplasia, but also with deformations of the proximal femur and femoral head. This condition, when left untreated, results in most cases in a rapidly progressing osteoarthritis, as early as in young adulthood or late adolescence, where a 4.3-fold increase in the rate of radiographic hip osteoarthritis is seen [24]. The untreated DDH is the most common cause of hip arthroplasty in young adulthood [17]. Beside genetic predisposition, DDH is the end result of a combination of intrinsic and extrinsic mechanical factors. Alongside a combination of morphological anomalies of the acetabulum regarding inclination, orientation, volume and size, DDH encompasses a series of malformations of the proximal femur and femoral head as well, consisting of insufficient femoral head coverage, abnormal version and excessive angulation from femoral shaft [17]. Dysplasia is solely an example of the numerous pathologies involving the proximal femoral morphology and that of the femoral head and causing devastating consequences in young adulthood or late adolescence, thus proving that the geometry of the proximal femur plays a fundamental role in the existence and progress of these conditions [4].

In order to detect such abnormalities, normal ranges of morphometric parameters of these structures have to be previously defined [5]. The anthropometry of the adult hip has already been described [35]. In children however, such studies hardly exist, and when they do, they are based on plain X-rays or two-dimensional computed tomography (2D CT), that do not necessarily reflect the most precise measurement method [25]. These methods show many limitations especially in cases where the position of the patient during imaging varies, in pelvic inclination or rotation for example [38]. Consequently, as recommended by many authors [19] and in order to deliver the most precise measurements, three-dimensional computed tomography (3D-CT)-based techniques were adopted in this study as cross-sectional imaging technique for more precise measurements without overlapping and true of scale.

Aim of this study was to provide standardized reference values at a broad age range—to the best of our knowledge unprecedented in the literature so far—concerning the anthropometric morphological development of the of the proximal femur and femoral head based on 3D-CT techniques in asymptomatic children/adolescents and highlight any deviations regarding side and gender.

## Material and methods

### Study population

In this retrospective study, an anonymized data base was used to measure the linear and volumetric dimensions of the proximal femur and femoral head in asymptomatic children < 15 years of age.

Analysed age groups were categorized according to the year of birth: the first year of life (from birth till 12 months of age) was referred to as group 0, children aged 13–24 months were categorized as group 1, children aged 25–36 months were categorized as group 2 etc.

Patients > 15 years or patients presenting with hip symptoms were excluded. Further exclusion criteria included tumours in the hip region, Perthes disease, slipped capital femoral epiphysis, destructive arthritis of the hip, hip dysplasia, hip deformity and previous hip surgery or hip trauma. All performed scans were clinically indicated and were done for non-hip-related reasons: 52/85 (61%) were trauma related scans and 33/85 (39%) were done on patients with abdominal symptoms. None of the CTs was done for the sole purpose of the study.

Prior to the performance of the measurements, all CT exams were read by a board-certified radiologist (SJ) and a board-certified orthopaedic surgeon (AD) in consensus evaluation to exclude any hip deformity or trauma and confirm the eligibility of the patients to be included in the normative collective. All included CT exams were then read by two orthopaedic surgeons (AD and AJ) with 10 and 7 years of experience in the hip region blinded to each other’s measurements.

### CT Data acquisition and reconstruction planes

All analysed scans included the pelvis and both proximal femurs and were performed between 09/2008 and 09/2019 on different CT scanner systems within our institution (2 × 192 slice dual-source CT, 2 × 128 slice dual-source CT, 16-slice single-source CT). Using a helical technique with pitch factors from 0.55 to 3 and collimations of 0.6 mm or 1.2 mm, imaging was done in a standard supine position. Before performing the study measurements all datasets were uniformly reoriented and reconstructed in parallel and orthogonal planes to the pelvic anatomical plane in order to standardize the measurements between the scans, regardless of the default reconstruction within the Picture Archiving and Communication System (PACS) and regardless of the patient´s positioning during data acquisition.

Volume CT dose index (CTDIvol) ranged from 0.18 to 74.89 mGy. The broad range is due to the fact that indications for CT were highly variable in the retrospective collective analyzed and so was the scan range along the *z*-axis. Slice thickness was ≤ 1.5 mm with an increment equal or smaller than the slice thickness.

All measurements were carried out on the the aycan^®^ workstation OsiriX (aycan Medical Systems^®^, Rochester, NY, USA). A detailed list of the performed measurements is to be found in Table 1.

**Table 1:** List of the performed measurements with description and plane configuration

Name of the measurement method	Unit	Figures describing the measurement method	Description of the measurement methods	Plane configuration
Covered femoral head volume (CFHV)	cm^3^	Figure 1a	Boundaries of the covered femoral head on the most cranial, most caudal and on every 2nd axial slice were identified. A line connecting the anterior and posterior acetabular rims was used to precisely determine the covered part of the femoral head by the acetabulum (yellow line). Using the brush tool (aycan^®^ workstation OsiriX), the area of the covered femoral head was manually filled on each slice (red area). The areas of the missing slices were generated using the integrated ROI (region of interest) function in aycan^®^ workstation OsiriX. The volume of the covered head by the acetabulum (cubic millimetres) was then calculated by adding all the areas [27, 32][21, 32]	Measurement: axial
Femur head extrusion index (FHEI)	%	Figure 2a	Index resulting from dividing the non-covered portion of the femur head by the total diameter of the femur head in the coronal plane. Three lines are drawn perpendicular to the horizontal axis: the first one goes through the most medial point of the femur head (red), the second through the most lateral part of the upper acetabular rim (blue) and the third one through the most lateral part of the femoral head (green). The non-covered portion of the femur head is defined as the distance between the second and the third line (purple). The total diameter of the femur head is defined as the distance between the first and the third line (turquoise) [30, 33], [26, 36]	Measurement: coronal Measurements are done on the coronal slice, in which the femoral head is most prominent Axial plane: The horizontal axis in the axial plane extends through both femur head centres Sagittal plane: both axes go through the centre of the femur head
Coronal alpha angle (CAA)	Degree	Figure 3a	The angle between the femoral neck axis line and a line connecting the centre of the femoral head with the point, where the asphericity on the lateral side of the femoral head begins (red angle) [34], [7]	
Lateral centre-edge angle (LCEA)	Degree	Figure 4a	The angle between the perpendicular to the intercapital centre line, which is a line connecting both femur head centres (identified as the centre of a best-fit circle (yellow circle) around the femur head) and a line extending from the femoral head centre and the most lateral part of the acetabular roof (red angle) [30, 35], [26, 31]	
Femoral head diameter (FHD)	cm	Figure 5a	Diameter (turquoise) of a best-fit circle around the femur head (yellow circle), [13]	
Anterior head-neck offset (AOS)	cm	Figure 5a	The distance (pink line) between two parallels to the femur neck axis; the first one tangential to the most anterior part of the femur neck (red) and the second one tangential to the most anterior part of the femur head (green), [20]	
Posterior head-neck offset (POS)	cm	Figure 5a	The distance (purple) between two parallels to the femur neck axis; the first one tangential to the most posterior part of the femur neck (blue) and the second one tangential to the most posterior part of the femur head (yellow), [20], [13]	
Femoral neck-shaft angle (FNSA)	Degree	Figure 6a	The angle between the femur shaft axis and the femur neck axis: Femur shaft axis: width of the femur shaft (purple) is marked at two levels. The middle point (green) of these two lines is then identified. The axis is defined as the line going through these 2 points Femur neck axis: the line passing through the middle of the neck at its narrowest point (width is represented with a purple line) and the centre of the femur head (yellow circle) [36], [12]	Measurement: coronal Axial plane: The horizontal axis extends through the femur neck axis Sagittal plane: The vertical axis extends through the femur neck axis

### Measurements

The first author (AD) performed all measurements twice at a minimum interval of two months. The final values presented in the current study and all further analysis was based on the mean values of these two readings. The same measurements were performed by the senior author (AJ) independently. Both observers were blinded to each other’s results. Both observers performed the measurement according to a well-defined plane configuration set a priori (Table 1). The two readings of the first observer were compared to assess the intrarater reliability. The readings of the first and the second observer were compared to evaluate the interrater reliability.

The performed measurements included covered femoral head volume (CFHV), femoral head diameter (FHD), femoral head extrusion index (FHEI), coronal alpha angle (CAA), lateral centre–edge angle (LCEA), anterior (AOS) and posterior head–neck offset (POS) and femoral neck-shaft angle (FNSA). Selection of the measurements was done according to their clinical relevance. The femoral head extrusion index and the lateral centre–edge angle estimate the weight-bearing surface of the hip [15]. On the other hand, the femoral neck-shaft angle is used to evaluate the morphology of the proximal femur. These measurements are some of the classical parameters used not only to detect hip deformity but also to plan complex three-dimensional osteotomies of the pelvis [30].

### Statistical analysis

Mean values and standard deviations (SD) were used to describe quantitative morphometric parameters. Because of the high number of performed correlations (see Table 3) the Bonferroni method was used to correct increased error rates, and the statistical significance was indicated by a *p* value of < 0.00017. 95% double-sided confidence intervals (CI) were calculated. Student’s *t* test was used to analyze parametric data and Wilcoxon rank sum-test to analyze nonparametric data. Pearson coefficients (*r*) were used to assess bivariate correlations to age, gender, and side. Cronbach`s alpha (*α*) was used to evaluate internal consistency (see Table 2). Intraclass correlation coefficients (ICCs) were used to perform inter- and intra-rater reliability analyses for two of the authors independently at different time points, with at least 14 days between the two measurements (see Table 2).

**Table 2:** Inter- and intra-rater reliability

	Interclass correlation coefficient	Intraclass correlation coefficient	Cronbach`s alpha
CFHV	0.994295311	0.999306778	0.997004976
FHEI	0.899975883	0.915842526	0.958388929
CAA	0.628273276	0.876483893	0.89921559
LCEA	0.93636497	0.964951454	0.975725275
FHD	0.9985045	0.997920487	0.99840382
AOS	0.963908374	0.967606165	0.990130578
POS	0.981470989	0.99275307	0.986155231
FNSA	0.985650124	0.992330095	0.993130774

Covered femoral head volume (CFHV), femur head extrusion index (FHEI), coronal alpha angle (CAA), lateral center–edge angle (LCEA), femoral head diameter (FHD), anterior head-neck offset (AOS), posterior head-neck offset (POS), femoral neck-shaft angle (FNSA)

A repeated ANOVA (procedure Proc Mixed) was used to assess the distribution of cases inside each age group by evaluating the number of available cases per month of birth in each year. Taking case dispersion in each age group into consideration, growth phases were presented using sextic polynomial curves.

GraphPad Prism 8 (Version 8.4.2, San Diego, CA, USA) was used to create graphs. Statistical analyses were performed using SPSS (Version 9, IBM, Chicago, IL, USA).

### Ethics approval

This study was approved by the Ethics Committee of clinical research at our institution (Ethics Committee II, University Medical Centre Mannheim, Medical Faculty Mannheim, Heidelberg University, Theodor-Kutzer-Ufer 1-3, 68167, Mannheim, Approval 2016-870R-MA) and performed in accordance with the local ethical standards and the principles of the 1964 Helsinki Declaration and its later amendments.

## Results

The series consisted of 170 hips (85 patients, 46 males and 39 females), with a median age of 6 ± 5 years (males 6 ± 5 years and females 8 ± 5 years) (range 0–15). In total, 16 groups were formed (age range 0–15) with a median of 12 children per age group (range 4–16 cases). Each age group represented a year of life. The distribution of cases inside every year or age group was also analysed. The analysis was done calculating the number of cases according to the month of birth inside each year. The distribution between years was not significantly different (*p* = 0.7891).

The inter- and intra-rater reliability was high (range ICC 0.8–0.99 Cronbach alpha 0.86–0.99).

### Covered femoral head volume (CFHV)

**Fig. 1:**
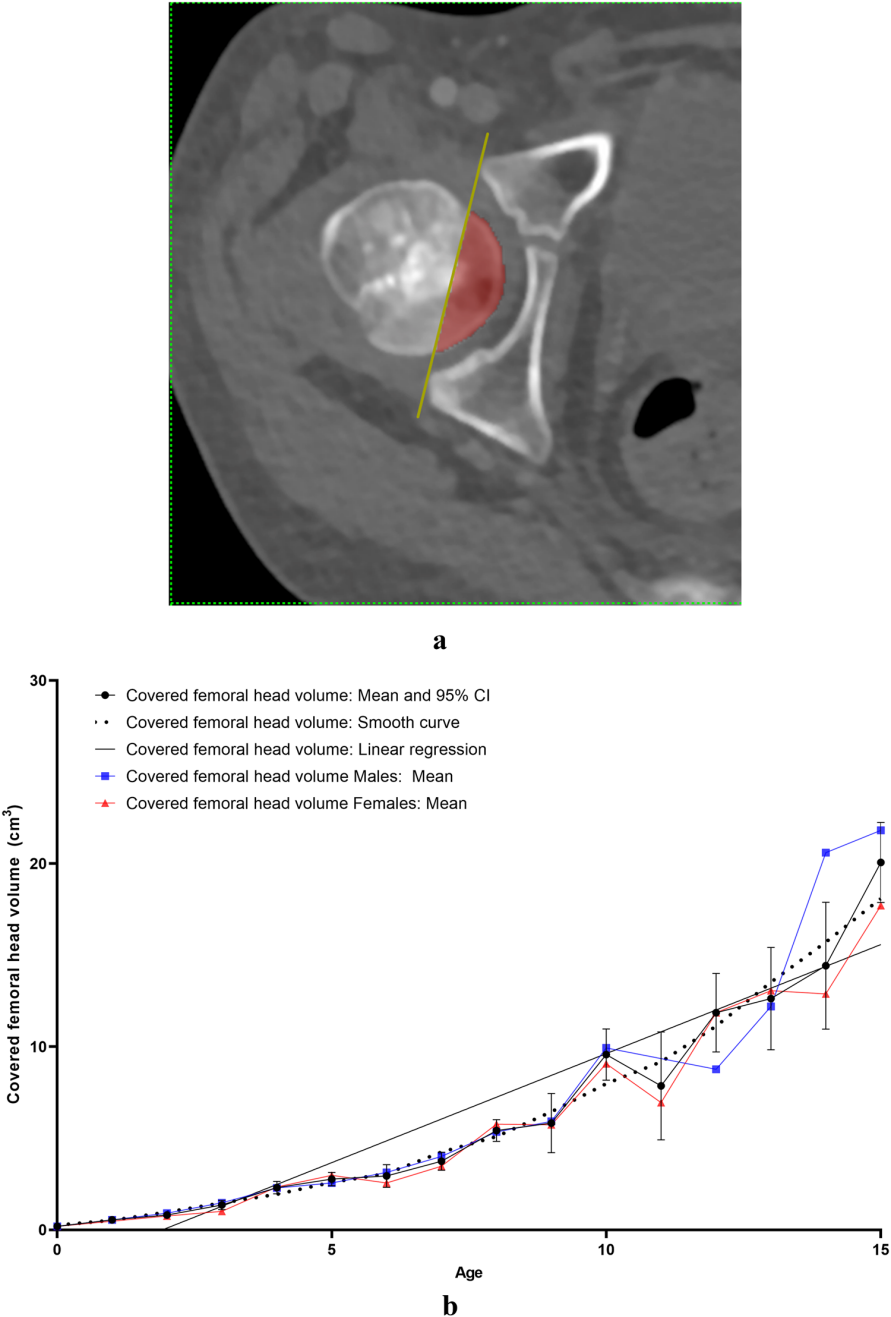
**a** Measurement method of covered femoral head volume (CFHV). *Red area: covered femoral head volume. **b** Results indicating the development of covered femoral head volume (CFHV) with age (colour figure online)

A significant high correlation between mean CFHV and age was noted (*r* = 0.893; *p* < 0.0001). CFHV increased from 0.2 ± 0.1 cm^3^ (males 0.2 ± 0.1 cm^3^ and females 0.2 ± 0.01 cm^3^) in children under 1 year of age to 20.1 ± 3.8 cm^3^ (males 21.8 ± 1.2 cm^3^ and females 17.7 ± 1.3 cm^3^) in 15-year-old patients (Fig. 1b). CFHV did not correlate with gender nor side. Prompt growth was noted at the age of 1 (0.19–0.55 cm^3^; 189%).

Further correlation analyses showed a significant high degree of correlation with LCEA (*r* = 0.792; *p* < 0.0001), ICEA (*r* = − 0.53; *p* < 0.0001), SASA (*r* = 0.791; *p* < 0.0001), DA (*r* = − 0.618; *p* < 0.0001), FHD (*r* = 0.878; *p* < 0.0001), AOS (*r* = 0.724; *p* < 0.0001) and POS (*r* = 0.873; *p* < 0.0001) (Table 3).

**Table 3:** Correlation analysis

	Gender	Age	Side	CFHV	FHEI	CAA	LCEA	FHD	AOS	POS	FNSA
CFHV											
*r*	0.515	< 0.0001	0.917	–	− 0.495	− 0.341	0.792	0.878	0.724	0.873	− 0.418
*p*				–	< 0.0001	< 0.0001	< 0.0001	< 0.0001	< 0.0001	< 0.0001	< 0.0001
FHEI											
*r*	0.226	< 0.0001	0.525	− 0.495	–	0.187	− 0.683	− 0.384	− 0.379	− 0.35	0.134
*p*				< 0.0001	–	0.015	< 0.0001	< 0.0001	< 0.0001	< 0.0001	0.082
CAA											
*r*	0.899	0.001	0.515	− 0.341	0.187	–	− 0.243	− 0.268	− 0.259	− 0.256	0.287
*p*				< 0.0001	0.015	–	0.001	< 0.0001	0.001	0.001	< 0.0001
LCEA											
*r*	0.138	< 0.0001	0.609	0.792	− 0.683	− 0.243	–	0.643	0.603	0.645	− 0.235
*p*				< 0.0001	< 0.0001	0.001	–	< 0.0001	< 0.0001	< 0.0001	0.002
FHD											
*r*	0.627	< 0.0001	0.975	0.878	− 0.384	− 0.268	0.643	–	0.812	0.895	− 0.596
*p*				< 0.0001	< 0.0001	< 0.0001	< 0.0001	–	< 0.0001	< 0.0001	< 0.0001
AOS											
*r*	0.017	< 0.0001	0.711	0.724	− 0.379	− 0.259	0.603	0.812	–	0.704	− 0.447
*p*				< 0.0001	< 0.0001	0.001	< 0.0001	< 0.0001	–	< 0.0001	< 0.0001
POS											
*r*	0.156	< 0.0001	0.626	0.873	− 0.35	− 0.256	0.654	0.895	0.704	–	− 0.479
*p*				< 0.0001	< 0.0001	0.001	< 0.0001	< 0.0001	< 0.0001	–	< 0.0001
FNSA											
*r*	0.228	< 0.0001	0.981	− 0.418	0.134	0.287	− 0.235	− 0.596	− 0.447	− 0.479	–
*p*				< 0.0001	0.082	< 0.0001	0.002	< 0.0001	< 0.0001	< 0.0001	–

Covered femoral head volume (CFHV), femur head extrusion index (FHEI), coronal alpha angle (CAA), lateral center–edge angle (LCEA), femoral head diameter (FHD), anterior head-neck offset (AOS), posterior head-neck offset (POS), femoral neck-shaft angle (FNSA), Pearson coefficient *r*, statistical significance *p* < 0.00017

### Femur head extrusion index (FHEI)

**Fig. 2:**
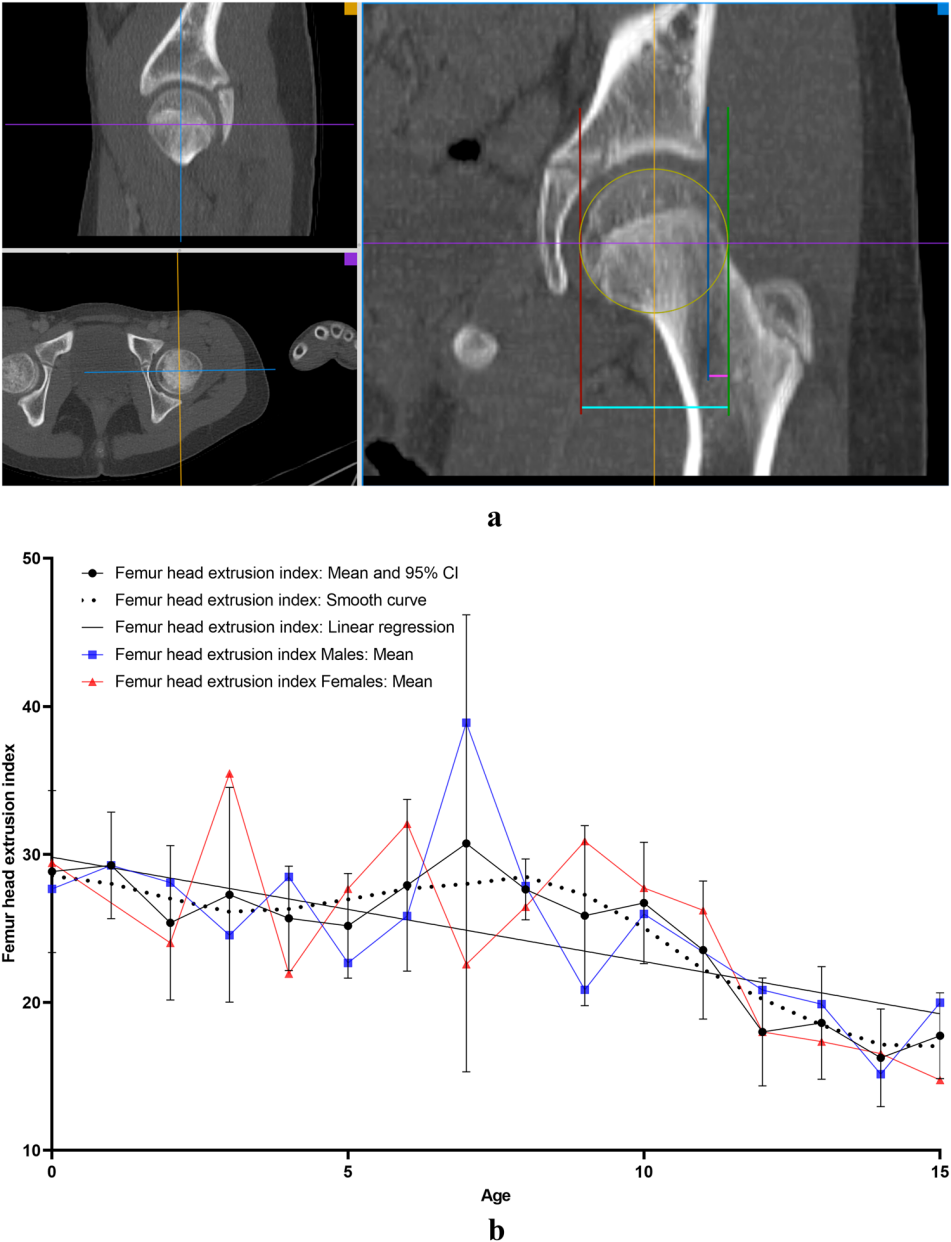
**a** Measurement method of femur head extrusion index (FHEI). *Uncovered part of femoral head (purple) divided by femoral head diameter (turquoise). **b** Results indicating the development of femur head extrusion index (FHEI) with age (colour figure online)

FHEI showed a declining trend with age (*r* = − 0.437; *p* < 0.0001) going from 28.9 ± 8.6% (males 27.7 ± 5.4% and females 29.4 ± 2.9%) in children under 1 year of age to 17.8 ± 5% (males 20 ± 1.7% and females 14.8 ± 1.6%) in 15-year-old patients (Fig. 2b). No correlation was found with gender or side. FHEI correlated negatively with LCEA (*r* = − 0.683; *p* < 0.0001) and SASA (*r* = − 0.683; *p* < 0.0001). Correlations with the remaining parameters were poor. Rapid growth phase was noted at the age of 11 (23.54–18.01; 23.49%) (Table 3).

### Coronal alpha angle (CAA)

**Fig. 3:**
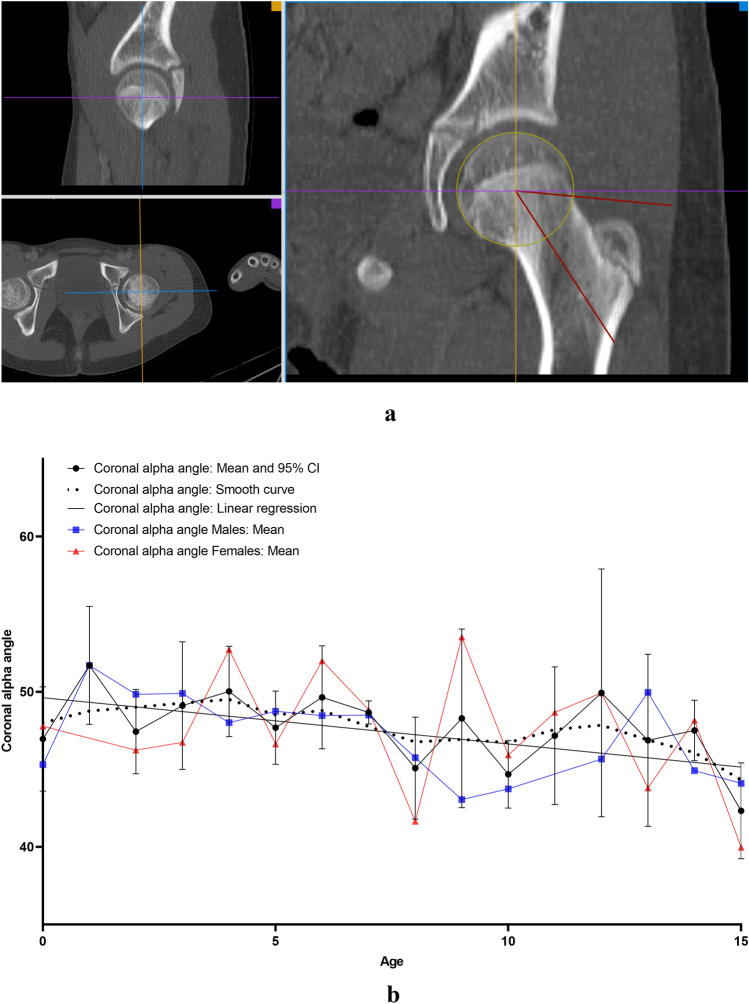
**a** Measurement method of coronal alpha angle (CAA). *Red angle: coronal alpha angle. **b** Results indicating the development of coronal alpha angle (CAA) with age (colour figure online)

CAA decreased with higher age (*r* = − 0.258; *p* = 0.001). The correlation with age was poor. Values of 47 ± 5.3° were observed in children under 1 year of age (males 45.3 ± 3.8° and females 47.8 ± 1.4°) and 42.3 ± 5.4° in 15-year-old patients (males 44.1 ± 1.4° and females 40 ± 2.6°) (Fig. 3b). No strong significant correlations were observed neither with gender/side nor with any of the other parameters. Rapid growth phase was noted at the age of 1 (51.69°–47.43°; 8.24%) (Table 3).

### Lateral centre-edge angle (LCEA)

**Fig. 4:**
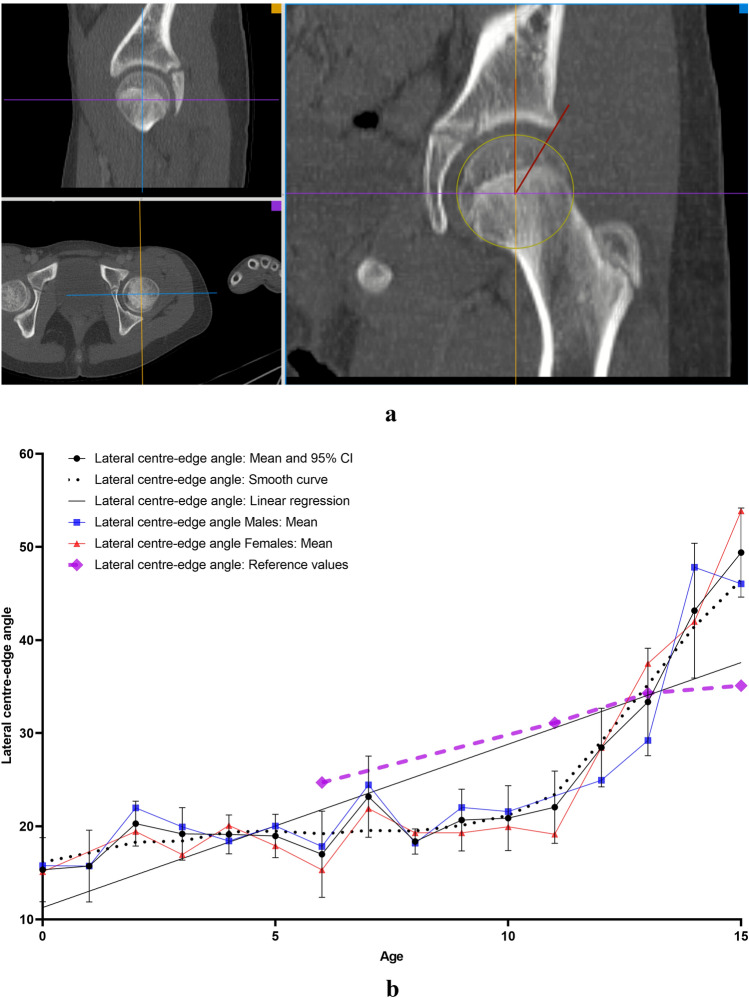
**a** Measurement method of lateral centre–edge angle (LCEA). *Red angle: lateral centre–edge angle. **b** Results indicating the development of lateral centre–edge angle (LCEA) with age. *Reference values: [29, 37] (colour figure online)

The LCEA (Fig. 4b) increased with age (*r* = 0.716; *p* < 0.0001). A strong significant correlation with gender or side was not found.

LCEA correlate significantly and strongly with CFHV (*r* = 0.792; *p* < 0.0001) and FHEI (*r* = − 0.683; *p* < 0.0001) as well as FHD (*r* = 0.643; *p* < 0.0001), AOS (*r* = 0.603; *p* < 0.0001) and POS (*r* = 0.645; *p* < 0.0001). Fast growth phases were noted at the age of 1 (15.72°–20.28°; 29%) and 7 (16.99°–23.18°; 36.43%) (Table 3).

### Femoral head diameter (FHD) and anterior/posterior head–neck offset (AOS/POS)

**Fig. 5:**
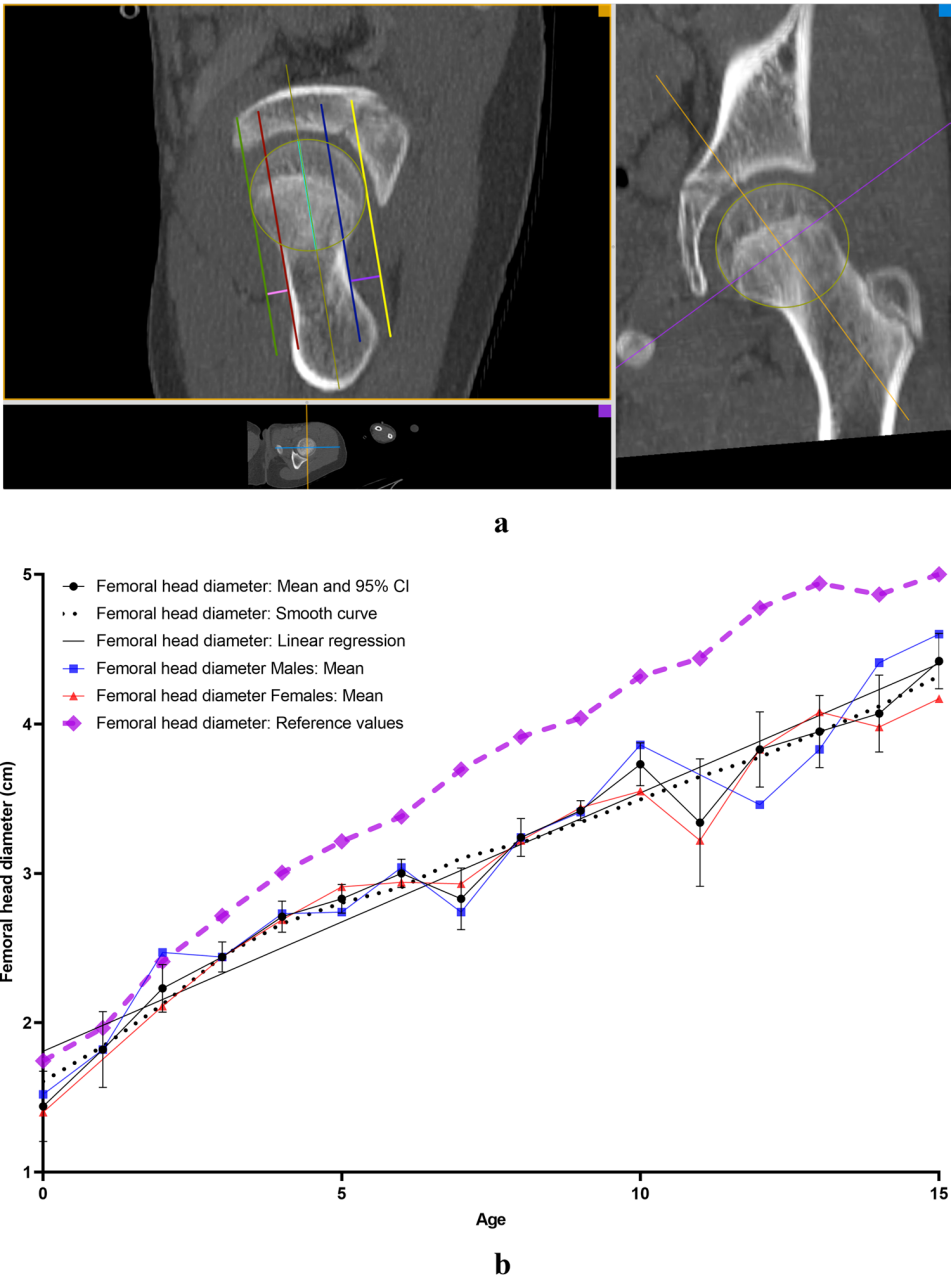

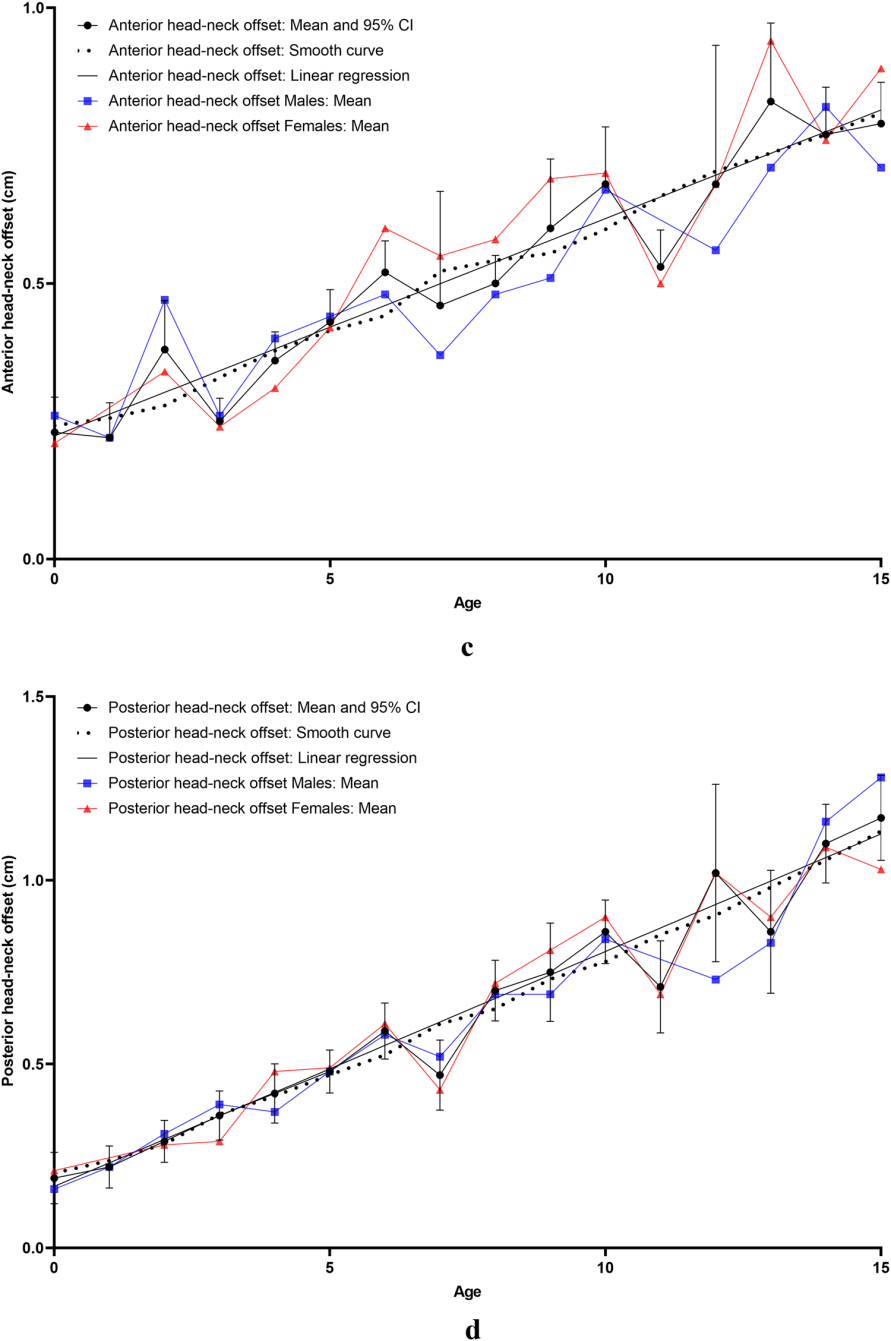
**a** Measurement method of femoral head diameter (FHD) and anterior/posterior head–neck offset (AOS/POS). *Turquoise: femoral head diameter. *Pink line: anterior head–neck offset. *Purple line: posterior head–neck offset. **b** Results indicating the development of femoral head diameter (FHD) with age. *Reference values: [35]. **c** Results indicating the development of anterior head–neck offset (AOS) with age. **d** Results indicating the development of posterior head–neck offset (POS) with age (colour figure online)

FHD (Fig. 5b), AOS (Fig. 5c) and POS (Fig. 5d) increased with age. The correlation was strong and significant (*r* = 0.931; *r* = 0.816 and *r* = 0.899; *p* < 0.0001). No correlation was found with gender or side.

FHD, AOS and POS correlated among each other as well as with CFHV (*r* = 0.878; *r* = 0.724; *r* = 0.873; *p* < 0.0001), LCEA (*r* = 0.643; *r* = 0.603; *r* = 0.654; *p* < 0.0001), SASA (*r* = 0.642; *r* = 0.601; *r* = 0.644; *p* < 0.0001) and DA (*r* = − 0.711; *r* = − 0.566; *r* = − 0.651; *p* < 0.0001). Fast growth phases were noted at the age of 1 (1.44–1.82 cm; 26.39%) for FHD and for AOS (0.22–0.38 cm; 72%). POS showed the fastest growth at the age of 7 (0.47–0.7 cm; 48.9%) (Table 3).

### Femoral neck-shaft angle (FNSA)

**Fig. 6:**
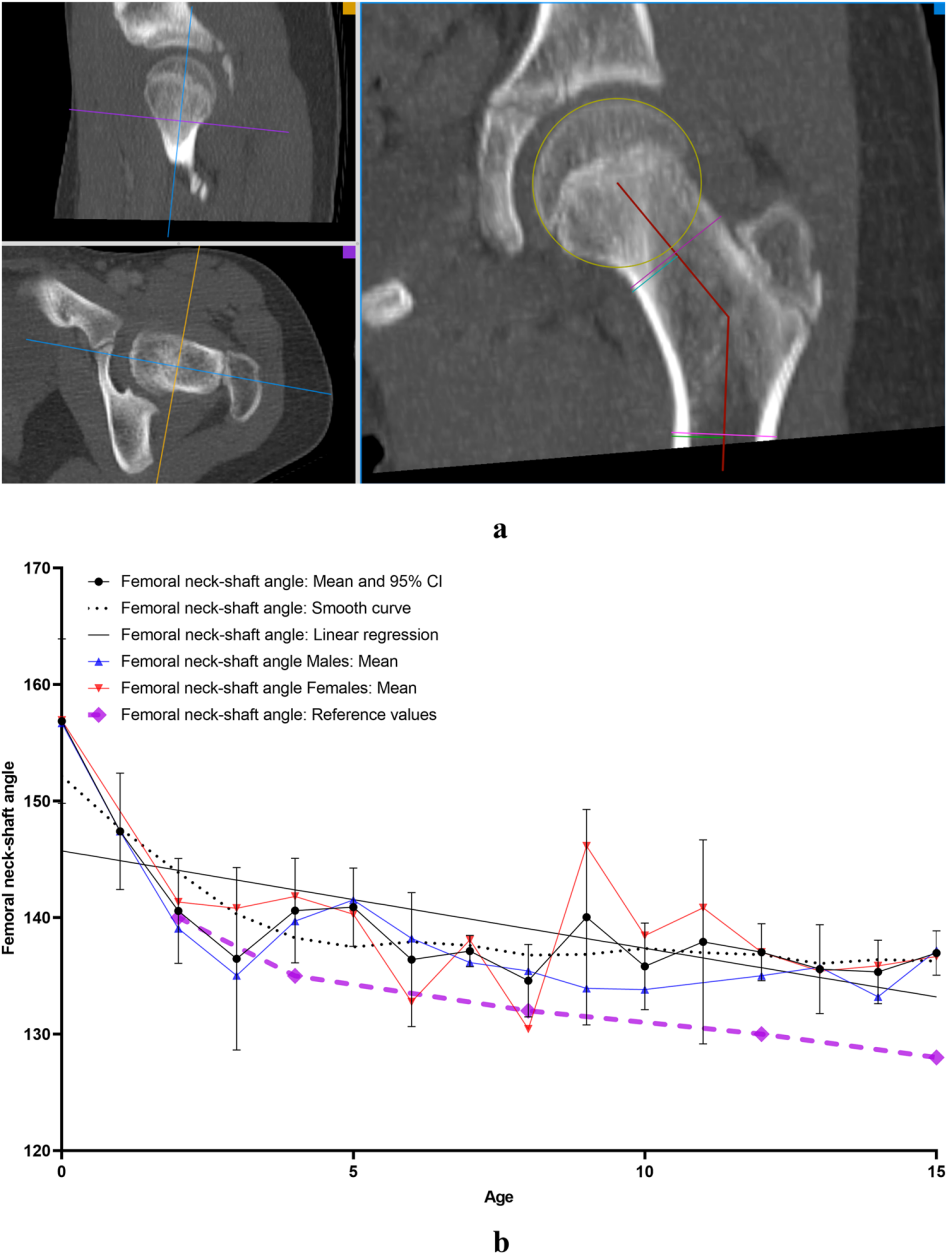
**a** Measurement method of femoral neck–shaft angle (FNSA). *Red angle: femoral neck–shaft angle. **b** Results indicating the development of femoral neck–shaft angle (FNSA) with age. *Reference values: [29] (colour figure online)

FNSA (Fig. 6b) angle showed a significant negative correlation with age (*r* = − 0.526; *p* < 0.0001) and FHD (*r* = − 0.596; *p* < 0.0001). No correlation with gender or side was found. Prompt growth phase was noted at the age of 1 (156.85°–147.4°; 6%) (Table 3).

Rapid growth phases were noted at the age of 1 for CFHV, CAA, LCEA, FHD, AOS and FNSA. A second growth phase was noted at the age of 7 with a prompt development of LCEA, and POS. A last growth phase was seen at the age of 11, where FHEI showed a marked increase.

A detailed list of the values of all measurements is to be found in Supplement proximal Femur.

## Discussion

In order to detect morphological abnormalities of the proximal femur and femoral head, normal ranges of morphometric parameters of these structures have to be previously defined. These ranges vary widely with growth and are specific to each age group [5]. The importance of these ranges is that they help reduce the number of missed pathologies and their consequences. For instance, functional scoliosis, limb length difference and back pain as well as gait anomalies, decreased strength and degenerative hip pathologies are just few of the problems that can appear in late adolescence or young adulthood in cases of late diagnosis of DDH [28]. In such cases of late diagnosis and because of the possible treatment complications, untreated children may even have a better clinical outcome as those treated too late [28].

Another issue is the imaging technique to be chosen in the measurement of these parameters. Plain radiographs were long considered to be the method of choice in evaluating hip pathologies especially when it comes to hip dysplasia in children [6]. Other authors prefer computed tomography (CT) scans because of their superior accuracy in comparison to conventional radiography [18].

However, even among skilled surgeons, all these two-dimensional techniques were shown to be insufficiently reliable when it comes to evaluating the extent of the morphological deformities [10], especially when a patient´s position during imaging varies like in cases of pelvis inclination or rotation or by deviation in the projection of the imaging technique [34]. Therefore, since almost all of the hip parameters depend on the pelvis position [6], many authors suggest 3D-CTs [19] to be the most precise technique in the measurement of such parameters, as it has been shown to be of superior accuracy compared to standard CT and plain radiographs [14] as well as of superior inter- and intra-rater concordance compared to 2D-CT [11].

Therefore, aim of the current study was to deliver standardized normal reference values of the radiographic parameters classically used in the daily clinical practice to detect hip deformity especially hip dysplasia and to compare them with the reference values already reported in the literature. Literature values are usually X-ray-based. The presented values are based on inclination and rotation-corrected computed tomography scans at a broad age range to describe the complete anthropometric development of the paediatric proximal femur.

In the present study a total mean FHEI of 24.7 ± 4.5% (± 1.1% standard error) was observed. The results correlate with the reference values for FHEI reported in the literature, where an FHEI of > 25% is considered as pathological [8, 23, 36]. Jandl et al. [9] measured the FHEI on the plain radiographs of 40 patients aged 5.8 ± 2.3 years (2–11). All included patients had a unilateral Legg–Calvé–Perthes disease. The measurements were performed on the healthy contralateral side. The mean FHEI was markedly lower with 7 ± 1.3% (standard error). Adjusting the ages of the patients of the current study to match those analysed in the publication of Jandl et al. [9] (2–11 years) would modify the mean FHEI to 26.6 ± 2% (± 0.6 standard error).

In the same study of Jandl et al. [9], the measurements were performed on the same 40 healthy hips using MRI scans. In comparison with the X-ray-based measurement an increase of the mean FHEI to 15 ± 0.7% (standard error) was observed. This increase is due to the cartilaginous portions that could only be evaluated on MRI scans. The result was still slightly lower as the values measured in the current study.

A mean LCEA value of 24.1 ± 9.9° was measured in this study. The results are comparable with those of Novais et al. [22] where values of 26 ± 5° in the CT scans of asymptomatic adolescents aged 10–17 years were measured but lower than the values presented by Tönnis et al. [29], where a mean angle of 31.3 ± 2.36° was measured.

Tönnis et al. [29] further categorized his X-ray-based reference values and considered LCEA values of > 20° for children aged 5–8 years and > 25° for children aged ≥ 9 years to be a sign of sufficient femoral head coverage. In this study LCEA values of 19.4 ± 2.7° were measured in children 5–8 years old and 31.1 ± 11.5° in children ≥ 9 years old. The first values would have been considered marginally pathological according to the reference values of Tönnis et al. [29]. This suggests that the X-ray-based techniques may overestimate the actual values of these measurements.

In the current study a mean FHD of 3.09 ± 0.87 cm in males and 3.13 ± 0.77 cm in females was measured. The values were slightly lower compared with those from the study of Wegener et al. [35]; here mean values of 3.74 ± 1.18 cm were measured in the radiographs of 323 male children and 3.58 ± 1.09 cm in 352 female children. All included children were under 16 years of age.

A total mean FNSA of 139.3 ± 5.6° was measured in this study. The values were slightly higher than the value 133 ± 2.1° presented by Tönnis et al. [29] but consistent with those from the study of Von Lantz et al. [33], where a mean FNSA of 136° was observed. Von Lantz et al. [33] further presented values reflecting the chronological development of the hip (FNSA 144° in children aged 1–3 years, 135° at 4–5 years, 134° at 9–13 years and 130° at 15–17 years). These values were fairly comparable and in part slightly lower compared with the values measured in the current study (FNSA 145.3 ± 8.9° in children aged 1–3 years, 140.8 ± 0.2° at 4–5 years, 137.3 ± 1.8° at 9–13 years and 136 ± 1.9° at 15 years).

It was not always possible to perform a direct comparison of our results with results in the literature. The limited publications found [9, 22, 33, 35] analyzed few or small age groups and included only some of the measurements. Jandl et al. [9] included 40 patients aged 5.8 ± 2.3 years (2–11), Novais et al. [22] 27 patients aged 13 ± 2 years (10–17) and Wegener et al. [35] 675 patients aged 9 months to 16 years. These studies examined the above-mentioned parameters and, therefore, only these parameters were compared to our results. The remaining measurements (CFHV, CAA, AOS/POS) were not compared with literature data, since no analogous studies measuring them in multiple age groups were found.

Regarding growth pattern, the measured parameters showed rapid growth phases at the age of 1, 7 and 11. These results correlate with those reported by Wegener et al. [35], who investigated the development of joint space growth and observed rapid growth rates at the age of 1, 8 and 12.

The included CT scans were performed for non-hip related reasons. This was not considered a limitation as the slice thickness in all analysed scans was of 1.5 mm or less, thus providing isotropic or nearly isotropic data that can be reconstructed after the imaging to any cutting plane.

One of the limitations of this study is the relatively small number of hips analysed in some age groups. The cases were also not distributed equally in all age groups, which may have made the charting of growth phases difficult. In addition, ethnicity was not recorded by the inclusion. In the metropolitan area where the study has been performed, the high ethnic variation could be considered as a limitation in the study.

The imaging techniques used for this study were performed in supine position. The absence of weight bearing while the CT scan is performed may have negatively affected the volumetric assessment of the covered femoral head [32].

Another limitation of the study is the underestimation of the cartilaginous structures in CT-based measurements. In order to avoid this, many authors suggest MRI-based techniques to address the inferiority of CT regarding evaluation of cartilaginous structures [22]. These techniques can provide a better assessment of cartilaginous parts; however, they showed many limitations. For instance, MRI techniques are not possible in patients with remaining metallic particles, due to artefact-related insufficient evaluation [2]. This problem is being currently addressed with the development of metal artefact suppression MRI sequences [27]. MRI-based measurements are also strongly affected by the variation in patients’ position during the scan and compared with standard radiography, MRI-based measurements showed high level of discrepancy [26]. Modern MRI sequences offer high in-plane resolutions but are not by default isotropic, which makes the modification or angulation of the cutting planes after performance of the imaging not possible. Stelzeneder et al. [26] compared the anterior centre-edge angle in conventional radiographs and in MRI. The latter showed markedly higher angles up to + 28° on both the sagittal and the oblique sagittal slices.

A last probable limitation may result from the fact that the patients included were not explicitly inspected for hip symptoms. All used CT scans were performed for non-hip-related reasons; however, it may indicate that the included cases may not represent a completely asymptomatic patient-collective.

At last, CT scans compared to MRI or plain radiography result in application of radiation or respectively higher radiation doses. This has to be taken into consideration, especially in young patients, as the stochastic risk of radiation-induced cancer or leukaemia development in this age group is higher. Thus, the importance of the correct CT indication, sticking to the A-L-A-R-A principle.

## Conclusions

This wide-ranging quantitative analysis of the anthropometry of the proximal femur and femoral head in children/adolescents under 15 years of age should be considered as a tool for paediatricians and orthopaedic/paediatric surgeons for early diagnosis of deformities in this area and provide guidance in the planning of possible operations.

## Supplementary Information

Below is the link to the electronic supplementary material.

Supplementary file1 (XLSX 18 KB)

Supplementary file2 (DOCX 253 KB)
