# Supplementary material for: Anthropometry of the proximal femur and femoral head in children/adolescents using three-dimensional computed tomography-based measurements
Source: Surg Radiol Anat. 2021 Oct 1;43(12):2009–23. doi: 10.1007/s00276-021-02841-3 (PMC8536629; doi:10.1007/s00276-021-02841-3)

**Sextic polynomial curves depicting growth phases**

Covered femoral head volume (CFHV)

Femur head extrusion index (FHEI)

Coronal alpha angle (CAA)

Lateral center-edge angle (LCEA)

Femoral head diameter (FHD)

Anterior head-neck offset (AOS)

Posterior head-neck offset (POS)

Femoral neck-shaft angle (FNSA)


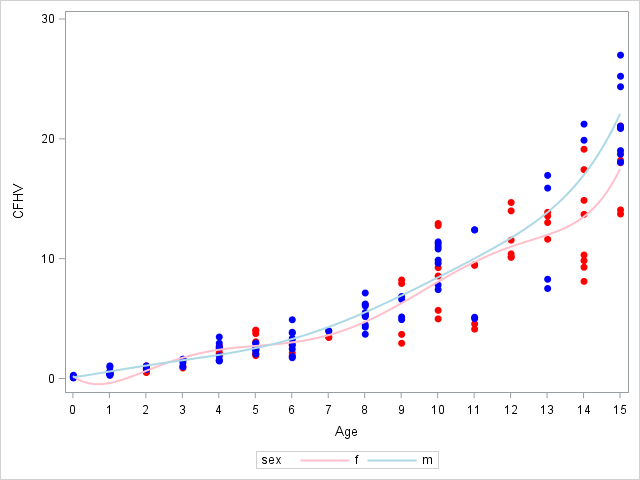

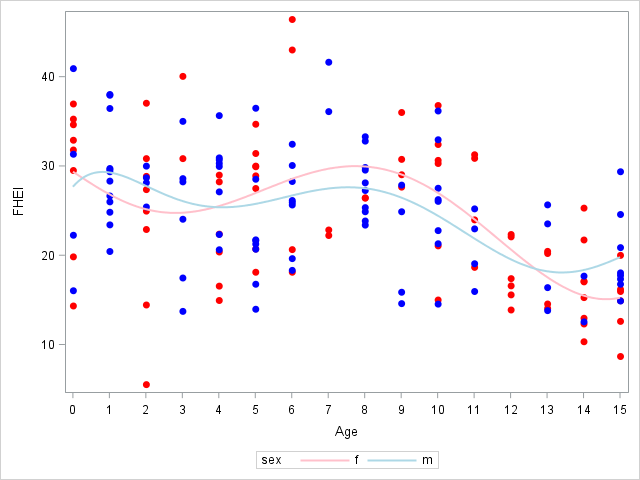

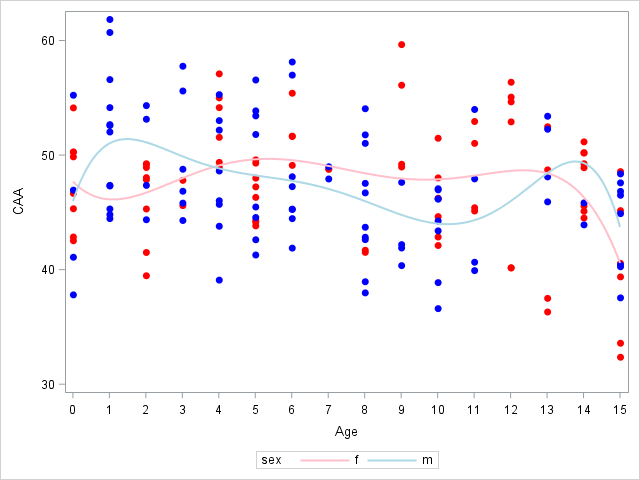

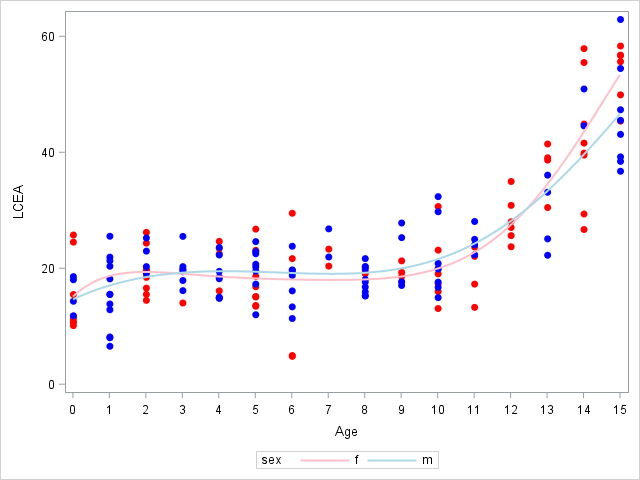

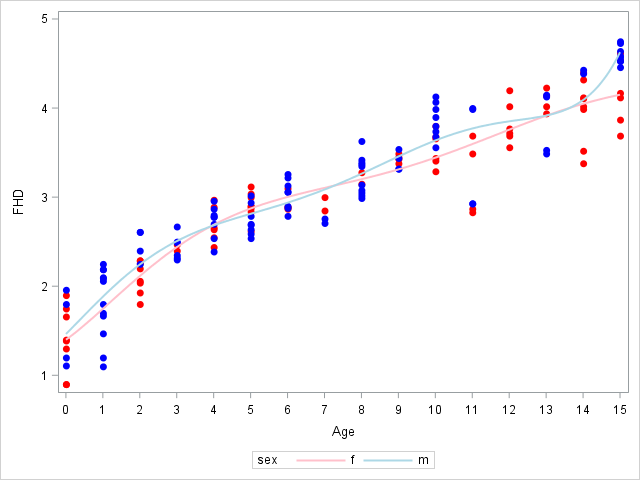

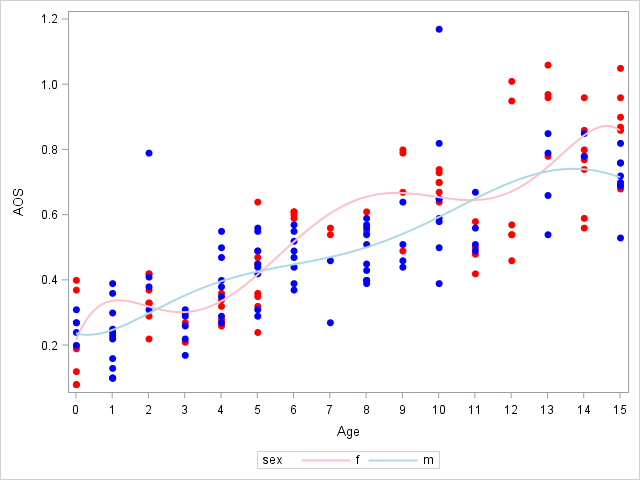

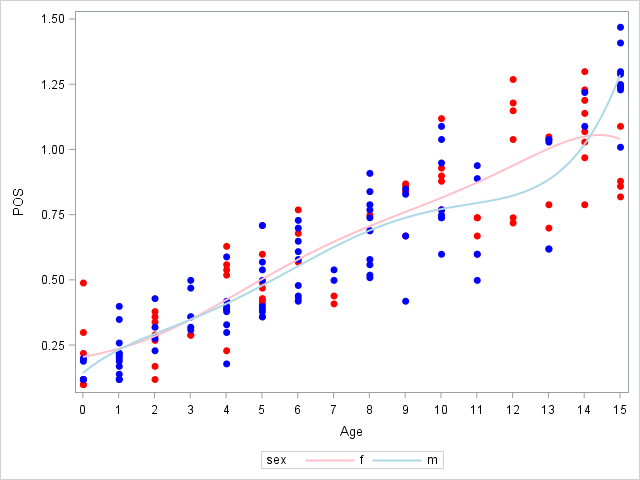

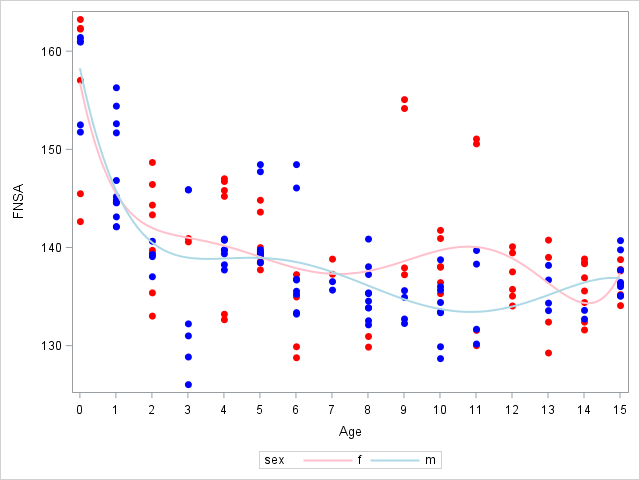

Supplement: Supplementary file 2 — Supplementary file2 (DOCX 253 KB) [file 276_2021_2841_MOESM2_ESM.docx]
